# A CMMI-based approach for medical software project life cycle study

**DOI:** 10.1186/2193-1801-2-266

**Published:** 2013-06-17

**Authors:** Jui-Jen Chen, Wu-Chen Su, Pei-Wen Wang, Hung-Chi Yen

**Affiliations:** Chang Gung Memorial Hospital, Kaohsiung Medical Center, Chang Gung University College of Medicine, No. 123, Da-Pei Rd, Niaosong Hsiang, Kaohsiung County, 83301 Taiwan ROC

**Keywords:** Nuclear medicine, Patient safety, CMMI, SysML, Project management

## Abstract

In terms of medical techniques, Taiwan has gained international recognition in recent years. However, the medical information system industry in Taiwan is still at a developing stage compared with the software industries in other nations. In addition, systematic development processes are indispensable elements of software development. They can help developers increase their productivity and efficiency and also avoid unnecessary risks arising during the development process. Thus, this paper presents an application of Light-Weight Capability Maturity Model Integration (LW-CMMI) to Chang Gung Medical Research Project (CMRP) in the Nuclear medicine field. This application was intended to integrate user requirements, system design and testing of software development processes into three layers (Domain, Concept and Instance) model. Then, expressing in structural System Modeling Language (SysML) diagrams and converts part of the manual effort necessary for project management maintenance into computational effort, for example: (semi-) automatic delivery of traceability management. In this application, it supports establishing artifacts of “requirement specification document”, “project execution plan document”, “system design document” and “system test document”, and can deliver a prototype of lightweight project management tool on the Nuclear Medicine software project. The results of this application can be a reference for other medical institutions in developing medical information systems and support of project management to achieve the aim of patient safety.

**Electronic supplementary material:**

The online version of this article (doi:10.1186/2193-1801-2-266) contains supplementary material, which is available to authorized users.

## Introduction

The main objective in this research is to provide precise and unambiguous specifications of the system requirement analysis, system design and testing, then establishing traceability between them. The traceability provides the foundation for clearly identifying the design aspects that are related to the requirements. This in turn allows software project owner to more effectively check whether the requirements are addressed by the design. Besides, there also has been a heated debate to discuss how to successfully develop Health Information System (HIS) with proper software engineering approach, such as development framework, requirement management methods in recent years. The reasons behind these methods are to achieve project success goals. For instance, an ineffectively requirement change management will impact schedule, cost and produce non-necessary redesign for project (Fu et al. 2012; Ißler et al. 2007; Wendt et al. 2004; Hasman & Haux 2006). Reference (Leonor Teixeira & Santos 2012) proposed a User-centered design approach, user can use interview or questionnaire throughout entire software project phases to make sure project success and avoid unnecessary cost and failure. Reference (Hasvold & Scholl 2011) pointed out software development should focus on interaction between user and technology, not only on particular side. Therefore, a social technical concept of Participatory Design (PD) was proposed. Reference (Hägglund et al. 2010) in their OLD@HOME project that used structured scenarios approach by both developers and users to capture healthcare professionals’ current work situations, as well as their expectations, needs and requirements. However, these approaches do not cover the other phases of project and lack of systematic way to follow the software development standards to manage the project. For example, in post-implementation phase, there may have differences on actions of project support based on the experience and knowledge of the involved healthcare professionals and the developers. Thus, without proper and standard management model, the system may become an obstacle or ineffective use, and may not be used easily for the relevant stakeholders and public. Given this background, the specific research questions addressed in the current study are:

1. How can the existing software engineering approaches, be incorporated/enhanced in the context of medical software project management?
2. How can the conceptual model be utilized effectively to facilitate management of traceability matrix in different project phases by multiple perspectives?

To address these research questions, this research introduced a novel approach for the support of (semi-) automatic maintenance, generation of traceability relations of artifact-based and collaboration views (Marcus et al. 2005) in the software project and integrated software engineering models and user requirements to express in structural System Modeling Language (SysML) (Group OM 2012) diagrams to convert part of the manual effort necessary for traceability maintenance into computational effort. There are important innovations with the approach: first is the integration of LW-CMMI model and stakeholders’ needs, then grouping identical concepts for ease of project design and maintaining effort. Besides, the concepts of purposed multilayer (Domain, Concept and Instance) approach, given academic researcher and industry practitioner a widely understanding of varied important factors in different phases of software project. Then, we applied this framework on CGMH (CHANG GUNG MEMORIAL HOSPITAL) medical research project to prove its feasibility and effectiveness.

The paper is organized as follows. The topics of software engineering approaches, traceability management and related research are discussed in Section 2. Section 3 discusses the details of conceptual model, outlining its scope in each phase. Sections 4 demonstrate the results of execution of lightweight support tool in medical research project. The paper concludes with some suggestions for future research in the area.

### Literature review

#### Software development standards in Health Information System (HIS) field and the categorized of current software engineering approaches

At present, there are several standards in health care domain, for example: ISO EN 13606–1, OpenEHR and HL7 CDA. However, most of them focus on EHR (Electronic health records). There are seldom medical software approaches to be mentioned for effectively management and development processes of HIS. The EN 12967 proposed an idea that based on RM-ODP framework to describe three system views (Enterprise, Information and Computational), but it does not provide any details for system development (Lopez & Blobel 2009). Therefore, this research tried to study the general software engineering approaches and developed a model to prove its efficiency and effectiveness to our research project.

On the other hand, there are three different software development models were categorized by (Magdaleno et al. 2012). They are agile development, plan-driven and FOSS (Free Open Source Software). And, they also emphasized proper software development should base on project type to use suitable model to achieve better productivity. There is no specified model for all kinds of projects. For example, the plan-driven model is typically exemplified by maturity models such as CMMI-DEV (CMMI Product Team 2010). In this scenario, the plan-driven model is categorized by its characters of detailed planning and focus on well-defined and continuous improved processes. However, these development standards may not fit for small and medium size projects and organizations. The reasons were organization was small, the services are too costly and the organization has no time and personnel (Staples et al. 2007).

#### Light-weight CMMI

To facilitate development of open source programs and cultivate domestic software developers, The Department of Engineering and Applied Science, National Science Council of Taiwan invited experts from both the academic and practical fields to form a research team and develop the LW-CMMI (Hong et al. 2004). The research team also proposed The Open Source Technology Research Plan and listed the primary focuses of the research. By introducing systematic software development processes, the team attempted to encourage academic research on open-source technology and development of key techniques and applications of open source codes. The Light-Weight CMMI process areas include project management and support. The project management process area includes requirements management (REQM), project planning (PP), project monitoring and control (PMC) and risk management (RSKM). The support process area includes configuration management (CM), Decision Analysis and Resolution (DAR), Project Integration (PI), measurement and analysis (MA) and process and product quality Assurance (PPQA). Compared with CMMI-DEV 1.3(CMMI Product Team 2010), the LW-CMMI model tries to meet some specified requirements that be considered in lightweight software project. In the same time, it also provides templates for each milestone. Although each of them does not have specified implementation details, but the project owners can follow the goals from the template and reference current renowned approaches to develop the system. Therefore, the software project management will have more flexibility, reduced learning curve and the cost. In this paper, we will adopt LW-CMMI software engineering approach as our system model requirements.

#### Influencing factors of successful HIS project

A summary by (Gagnon et al. 2012) indicates that there are several factors which have huge impacts on successful implementation of health IT project. It includes: user participation at project development and implementation phases, defining right personnel for task assignments, providing suitable training and support based on knowledge and skill needs of tasks, and monitoring system use in the early stages of implementation (Gagnon et al. 2012). In addition, in order to pave the way of implementing Electronic health record (EHR). Meidani et al. pointed out that we should go far beyond technical perspectives and focus on organizational and managerial factors in quality improvement (Meidani et al. 2012). Thus, organization must continiuously improve their business process, team building and user involvement, innovation, change management by varied management technologies, such as Business process improvement (BPI). Business process reengineering (BPR) ( Meidani et al. 2012). Ting et al. (Ting et al. 2011) also provide twenty three critical elements about successful implementation of RFID-based health management projects in medical organization. These essential elements arecategorized at the project phases of preparation, implementation and maintenance and reveal technological, economic and operational perspectives of their approach (Ting et al. 2011). Thus, the well-planning, well-defined and continuous improved processes for successful ICT project management in medical organization are necessary of patient safety. In addition, the proper framework should have capabilities to put considerations on different theories and approaches. Because we know there should have more and more researches to be conducted for achieving project success based on varied management technologies (Meidani et al. 2012).

#### Traceability management

An approach to support the automated update of traceability relations between requirements, analysis and design models of software systems and express in the UML was purposed and made possible by analyzing change events that have been captured while working within a third-party UML modeling toolby (Mäder & Gotel 2012). However, traceability links between these project artifactsmay face problems to achieve traceability over different organizationsand varied modeling tools and also report problems in linking the gap between specification and design. As we know, there tend to be more and more global software projects are established due to advancement of Internet technologies. For example, software outsourcing and free open source software projects. Thus, the efficient ways are needed to manage the projects in current complex software development environment. Precise management model for facilitating the knowledge and traceability exchange between the boundaries of tools and organizations was a considerable problem.

In addition, (Soares et al.^2011^) pointed out that traceability management helps us to ensure that all requirements are fulfilled by the system and subsystem components. When the design elements are not completely followed requirements, there has some chances to lose focus as to the specific functions of each design blocks which leads to huge changed cost for rework and incorrect or missing functionality in delivered project. One way to manage the requirements traceability in SysML is by using requirement tables. SysML allows the representation of requirements, their properties and relationships in a tabular format as shown in Table 1 (Soares et al. 2011).

**Table 1:** **Requirements relationship table of SysML**

Id	Name	RelatesTo	RelatesHow	Type
T1	External common	T3	DeriveReqt	External
T2	Acceptance	T1	Satisfy	Non-functional

Therefore, this study integrated widely acceptance modeling tools-MS Visio and MS excel (VBA) to overcome the integration issues with third-party modeling tool for project management approach.

## Material and methods

**Figure 1:**
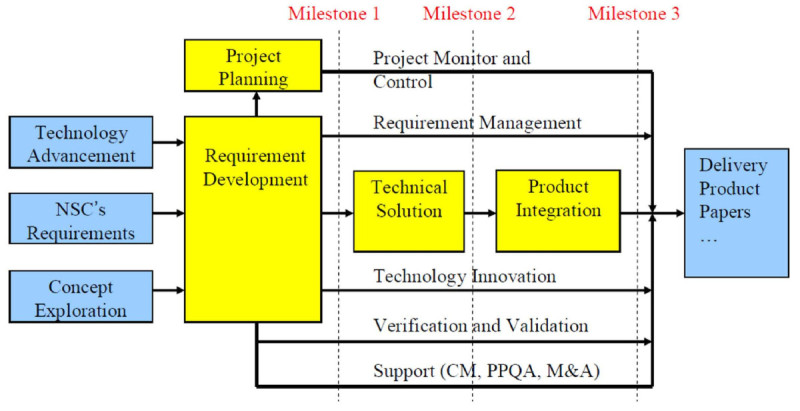
**Life cycle of software project development.**

**Figure 2:**
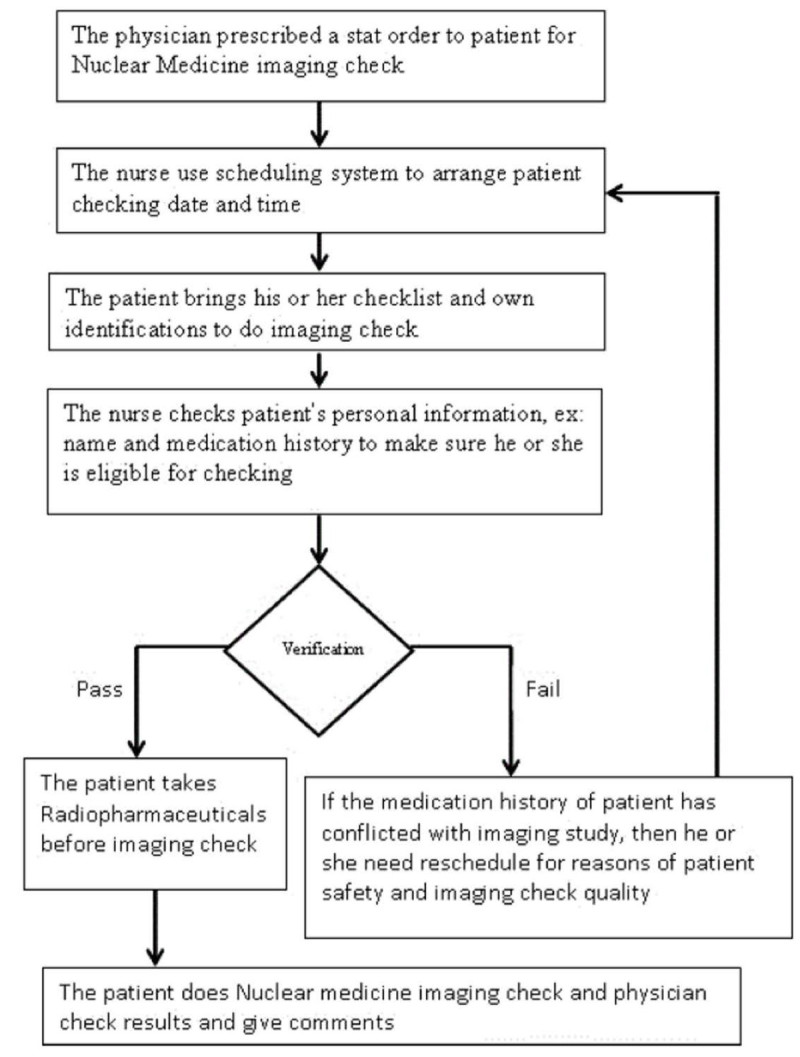
**Nuclear medicine imaging check workflow for patient.**

The proposed methodology employed LW-CMMI (Hong et al. 2004) as system development model. The development model can be seem as system requirements and has multiple phases. For instance, in milestone1: requirement analysis and project planning, milestone2: solution exploration and system design and milestone3: system implementation, integration testing and delivery. The life cycle was listed in Figure 1. And, these system requirements have their own artifacts. For example, requirement specification reviews and project execution plan reviews are placed in milestone1. And, the rest are system design document reviews and system test plan document reviews in mlestone2. In the same time, the workflow for Nuclear medicine imaging check process for patient is listed in Figure 2. We will describe operation flow, and then give user requirements list first. After that, we will give the LW-CMMI system requirement lists and present the integration of them into three layers model with standard software engineering approach to provide reference methodology for academic researcher and practical developer from industry to support project management. The details are listed as bellows:

### Patient Nuclear medicine imaging check workflow

While the other medical examinations without enough information for judging the situation of patient. Nuclear medicine imaging check is used for medical auxiliary examination report. Thus, we need some checkingmechanisms to guarantee the patientsafety and the quality of imaging check. Before implementation of our new system, the nurses need to schedule tasksmanually and ask patient about medication history before doing the imaging check. But, these actions may be ineffective and costly. In addition,the physicians also need auxiliary informationto help them for checking imaging results.For example, the reading materials and the sharingexperiences fromthe seniors and their colleagues. Therefore, a new web-based system (MHSAS) was designed (Chen et al. 2012) for ease ofoperation flow and time.

The system services were defined and gathered from different methodizes based on workflow in Figure 2, ex: observations, interviews and the other reading materials. Thus, it would provide the following functions:

- The system can provide support to gather patient medication history from database regularly for nurses. The data should include chart no, name, medication start date and end date.
- The user interface for query should include columns of chart no and schedule date, check date, check item and medication name.
- The system must provide suggestions for nurses and physicians when comparisons medication history and Nuclear medicine checking rules (ontology).
- When patient’s medication history will affect imaging quality, the alert system can prompt messages to provide warnings for the physicians.

### Light-weight CMMI requirements

1. Requirement Management Specification
  It was designed to meet REQM process area in CMMI. The requirements for the documents should define its purpose and acceptance criteria, such as clearly and properly stated, completely, consistently, uniquely identified, appropriately implement and verifiably. And, all of requirements must define their priority. Furthermore, the contents also should include operational concepts and requirements lists. In our research, the Use case diagrams from SysML were employed to show operation concept to meet the criteria.
2. System Design Requirements
  Using functional and interface requirements from requirement specification stage to develop system architecture and ER-Module diagram. Furthermore, each system has its own characteristics and needs. Therefore, we can use these screening criteria to decide technical solution. The listed diagrams in the SD document were Class diagram, Sequence diagram and State diagram.
3. Project Execution Plan Requirements
  In this document, it integrated CMMI process area, such as PP, CM, M&A, DAR, PMC and PPQA. Then, we followed and referred the rules that were listed in the requirements to facilitate the achievements of the project goals.
  3.1) Work Breakdown and Items Estimation Model
  The entire system development project was divided into project management, system engineering, software development, system integration and testing, software and hardware procurement and project support. Firstly, in order to describe each task under specified stages, we can use WBS (Work Breakdown Structure) and list full work packages and their tasks. In the breakdown of majority works, the size and complexity of each work item were estimated based on Line of Code, Number of Logic Gates, and Number of Pages. The other items were estimated based on researchers’ personal expertise and experiences. Secondly, The attributes of each task have task no, name, working artifacts, complexity and its size, relevancies with other working items, the skills, knowledge and resources (hardware and software) for this task, delivery flag to customer, start date, end date and total working hours. Each task can base on experiences of experts to calculate personnel needs; the criteria should have complexity of tasks and available working hours for potential project members. The output should be skills, knowledge of tasks and its estimated personnel. After that, we can base on these shortages to deliver training plans. It should include training items, estimated training time, personnel and cost. Lastly, the personnel in the project should define their roles and tasks. Furthermore, it also should have human resource management plan for personnel changed. In addition, the project also should have resource plan based on task items to estimate its cost. And, the monitor frequency for the budget (e.g. monthly) and the solutions when the budget is overused are also should be mentioned.
  3.2) Project Life Cycle and Milestone Checking
  As we have confirmed all the techniques and specifications required for system development, we adopted the Waterfall Model for developing the system. This model consists of four major phases: Requirements Specification, System Development, System Integration and Testing, and Project Close-out. And, each of them should define the milestone to monitor progress of project phases.
  3.3) Data Management and Configuration Management Plan
  In this research, the management of privacy information in the HIS was our considerable point. Thus, the details of data management and storage methods for the project were reviewed and proposed as follows. (1) Source codes: all the programs are stored in a server. The researchers back up the storage once a month. One copy will be delivered to the project manager, and one will be kept by the researchers. (2)Electronic files and execution files: Electronic files and execution files are stored in removable disks. The researchers back up the files once a month. Both the project manager and the researchers will keep one backup copy of the files. (3) Emails related to the project: The name of the project is added to each email related to the project. A carbon copy (cc) of each email related to the project will be forwarded to the project computer technician for backup. (4) Paper document: The paper document is kept by the researchers. Secondly, the goal of configuration management is to “build” and “maintain” consistency in works. To achieve this goal, we suggested taking the following measures: Configuration identification, Configuration control, Configuration status accounting, and Configuration audits. In this research, these measures were assisted by free open source CVS tool (Concurrent Versions System 2012).
  3.4) Risk Assessment
  Any risk factors may induce project fail should be listed as well as Table 2.

**Table 2:** **Risk assessment example**

Priority	Risk item	Probability	Impact	Note
1	Insufficient staff training	30%	High	Reinforce training, advanced study, self-learning
  3.5) Measurement and Analysis Plan
  The measurement and analysis plan were intended to collect various types of data related to the project for analysis.
  3.6) PPQA Plan
  In order to make sure product quality, some actions and measurements are taken during the project. The plan should (1) objectively evaluate process and working process and define review process to make sure artifacts from each project phase are met to design specification. (2) Good communication between project owner, user, developer regularly to make sure user’s satisfaction and progress of each issue. (3) The problems and its solutions need to be recorded exactly for future references and analysis. Most importantly, project should assign personnel to do quality control and coordination in the project.
4. System testing requirements
  The testing plan should define scope, testing procedure and its acceptance criteria. The process and testing cases should have personnel, hardware and software specification, test data source and schedule. Furthermore, the attributes for testing cases should have id, name, testing target, relevance with other functions, priority, and testing scenario, expected results. Lastly, the test cases should include integration and acceptance testing cases. The testing results should be noted for pass/fail/incomplete after verification and validation. It was designed to meet CMMI PI process area.

### The DCI model and its implementation concepts

The DCI model was designed by three layers (Domain, Concept and Instance) to represent the concepts from analysis, design and testing phases during the software project.

**Figure 3:**
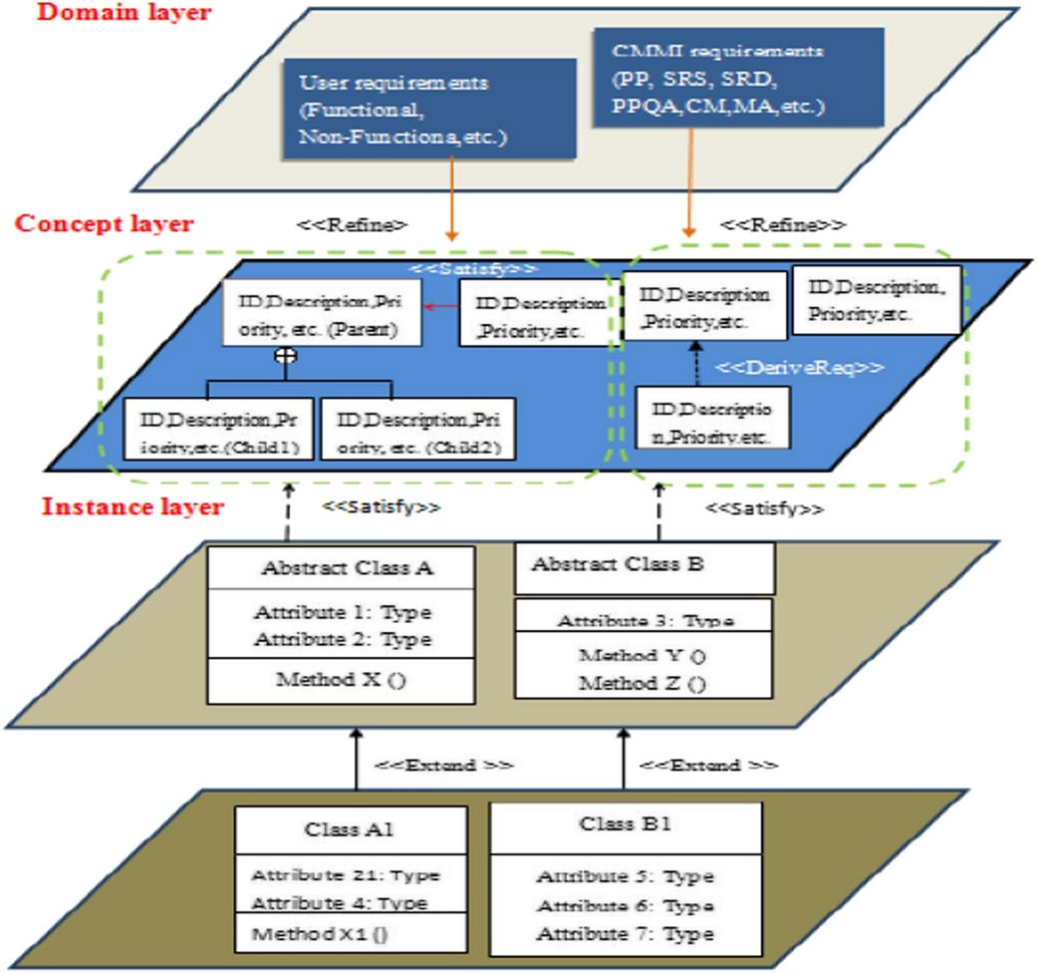
**The DCI model.**

The implementation concepts derived from Figure 3 are (1) Domain layer: the inputs of methodology may include software engineering standards and stakeholders’ requirements. (2) Concept layer: the input comes from the results of domain layer and uses characters of SysML to group requirements as the sub-packages. We based on needs and requirement types to present diagrams and their relations. In addition, the requirement has its own name and id. (3) Instance layer: the instance layer is represented for design and testing concepts. In order to ease of design and maintain effort, we summarized some common attributes from requirements as the parent classes. Then, the child classes can be built and inherited from their parent classes. In addition, we also put the test cases to verify and validate the functional requirements in this layer.

### Verification and validation rules

As shown in our modeling concepts, the supported tool will input data from third-party UML and SysML tools into our purposed approach. Thus, we assume the diagrams from the conceptual model will be correct in the analysis stage of project management. However, in some cases, it will have some unexpected reasons to induce massive errors if the original sources are incorrect. In order to avoid such kind of problems, we designed the general rules to verify diagrams during the initial phase of using the tool. Firstly, the requirements, the test cases and design classes in the project should be verified for its scope. If the requirement belongs to specified LW-CMMI process area and is linked to incorrect one. Then it will be deemed as an error. Secondly, the incorrect information is in the requirement. For example: name (type) and priority will be the evaluation factors for possible link errors. For instances, if the requirement type is functional or non-functional. However, its attribute is defined for opposite one. Then, it will be classified an error. Moreover, the priority for the requirement is important, because it is designed to meet project schedule. Thus, for non-functional requirement, in our project, it will not have highest priority, if its priority as highest (1), then we will say it is an error under verification criteria. In the current stage, we try to identify and list all possible checking rules in our project, and do verifications before the input of diagram data into our tool to make sure the correctness of system information. The demonstrated examples in our project are listed in the rest of section.

In the Table 3, we used the name of the artifacts in the different project phases (requirements, classes and test cases) to do verification. Thus, the test case (T1) should be linked to T2 (User-Interface requirement). But, it was linked to T3 (Performance requirement; wrong assigned type). In addition, the requirement (T4), it belongs to functional requirement and should have highest priority as 1 in our project. However, it was assigned to priority 2. Thus, we will deem it’s an assigned error. Lastly, the given design class (T5) was designed for CM (Configuration Management). So, in the instance layer, its concept must fulfill the requirement CM (T6). But, we can find it relates to T3 (Performance). Thus, an unexpected link error was happen.

**Table 3:** **Example of linked type errors**

Id	Name	RelatesTo	RelatesHow	Type	Priority
T1	UI_Test1	T3	Satisfy	Test case	N/A
T2	User-Interface	Null	Null	Non-Functional	1
T3	Performance	T4	Null	Non-functional	1
T4	Functional	Null	Null	Functional	2
T5	CM_Class	T3	Satisfy	Design class	N/A
T6	CM	Null	Null	Functional	1

After defining the conceptual model and testified rules, we can use them to effectively realize project management in different phases of project to consider varied success factors of implementation of CGMH medical software project which was approved by Institutional Review Board/ Chang Gung Memorial Hospital (IRB/CGMH) with reference number 100- 2903A3.

## Results

In this section, we present a study which applied three layers (Domain, Concept and Instance) modeling methodology on the medical software project. Firstly, we will describe the case background and the critical issues that justify the application of proposed approach. Then we will explain the study design, namely the methods and procedures of applying the principles project management process. After that, we present the results of the methodology process.

### Research case background

In order to meet the people’s growing demands for medical services, CHANG GUNG MEDICAL FOUNDATION with hospitals were built in Keelung and Kaohsiung following those in Taipei &Linkou, Taiwan. Besides, Chang-Gung Institute of Nursing and Chang-Gung University were established in the same time. The University and the Hospital work effectively together by complementing each other and its collaboration facilitate both basic research and clinical medicine (Chang Gung Medical Foundation 2012). Thus, the practical medical problems are encouraged to be researched for the well-being of public. The presented case was requested and studied by Nuclear medicine department of Kaohsiung branch. It is a 2600-bed-capacity medical center. In addition, the current HIS systems are dispersed in different locations, the physician and nurse need spent lots of efforts to integrate all necessary information to meet their needs. Therefore, from 2008, the related medical research projects have kicked off to resolve this issue. The prototype MHSAS was built as a web-based system. The system consists of four modules, including Medication History Collect Agent (MHCA), Medication History Search System (MHSS), Patient Medication Alert System (PMAS) and Patient Medication Consultation System (PMCS). The general operating process of these modules was explained in (Chen et al. 2012). In the same time, the systematic project management processes were agreed with all staffs in the department in order to achieve long term development goal. Thus, the research project participators were formed by physicians, nurses, IT staffs and support research staffs in the department, then joined through the whole project. In the below section, we will see the project artifacts in different phases and the demonstrated project management support tool for traceability management for requirements, design and testing in CGMH research project.

### Applying DCI model on CGMH project

**Figure 4:**
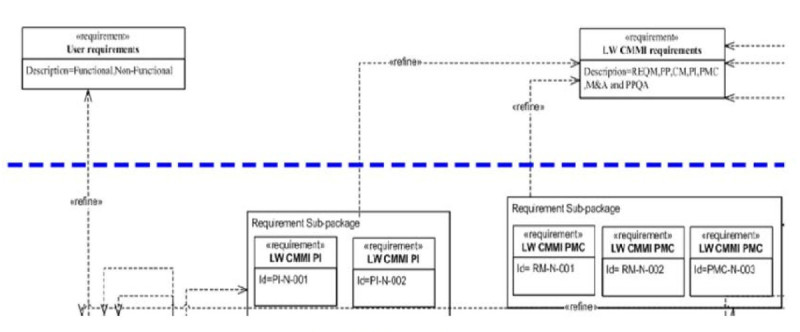
**Domain layer.**

**Figure 5:**
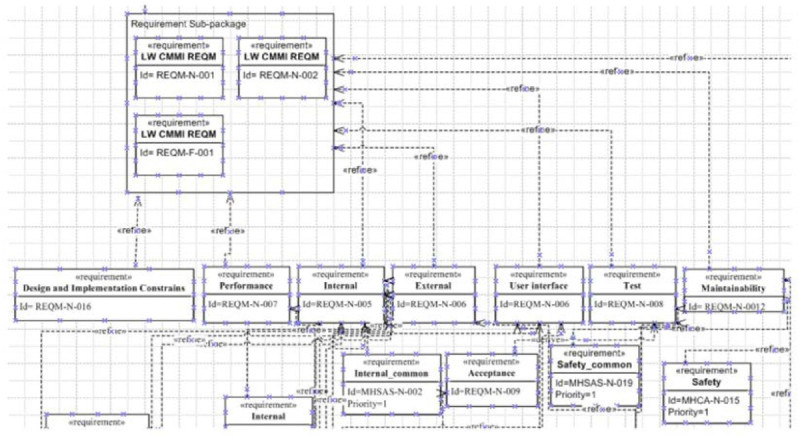
**An example of REQM process area in the concept layer.**

**Figure 6:**
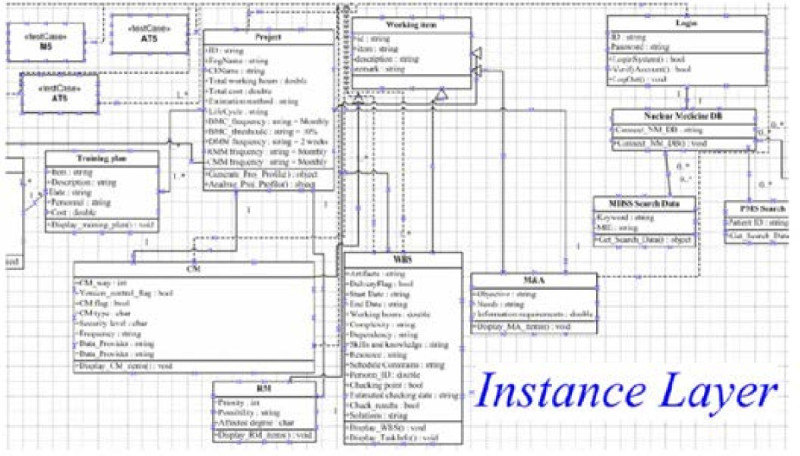
**System design and testing concepts.**

The Light-Weight CMMI and user requirements were prepared by project owner with various methodologies to be the inputs for the model, and some relevant and identical concepts are refined from domain layer as sub-packages in concept layer. Based on these requirements to derive design classes and testing cases in the instance layer for final stages of design and testing. The each layer of CGMH project diagrams are shown in Figures 4, 5 and 6.

1. Domain layer

The inputs are (1) the Light-Weight CMMI standards (2) analysis of relevant stakeholders’ requirements. As we can see from Figure 4, there are summary of major concepts (e.g. user and CMMI requirements) of project.

2) Concept Layer

As a result of input from domain layer, each sub-package represents individual process area in the Light-Weight CMMI. As pervious discussion, it does not provide any details for implementation. Thus, we refined implementation details from these goals by key process improvement areas to meet our project needs. For example, in the Table 4, we can see an example of REQM process area requirements (goals) and their summaries.

**Table 4:** **CMMI REQM requirements**

No	Description
REQM-N-001	The requirements should base on project purposes to define needs from users and functions. All processes should have control documents.
REQM-N-002	The control documents should have acceptance criteria, notation description and priority definition.
REQM-F-001	The system introduction should have description, operational concept and its requirements list. The major types of requirement are interface, functional, non-functional, performance, test and others.

Thus, based on these requirements, we can derive REQM sub-package and refine them by our needs with specific requirement type, for example: performance, internal, external, etc. Furthermore, the refined requirements must assign their priority to meet project schedule. The results are shown in Figure 5.

3) Instance layer

We summarized common attributes from user and Light-Weight CMMI requirements as ‘Working item’ class. And, CM, RM, WBS and M&A classes are inherited from ‘Working item’ class. In addition, test cases are designed to meet functional requirements in our research project.

### Traceability matrix management

In order to do change management, the tool can support management of traceability matrix tables from different prospects, such us artifact-based view and collaboration view based on our pervious discussion. User can export information of SysML diagrams from third-party tools, ex: Microsoft Visio. Then, the system can generate traceability matrix by user‘s requests into Microsoft Excel. Tables 5, 6 and 7 were the processed results by supported tool.

**Table 5:** **The exported information by SysML tool**

Id	Name	RelatesTo	RelatesHow	Type
T1	External common	{T3,T5}	DeriveReqt	Functional
T2	Acceptance	T4	Satisfy	Non-Functional
T3	Safety	T1	DeriveReqt	Non-Functional
T4	Internal common	T2	DeriveReqt	Functional
T5	Internal common	T7	DeriveReqt	Functional
T6	External common	T7	DeriveReqt	Functional
T7	User-Interface	{T8,T9}	DeriveReqt	Non-Functional
T8	Test	{T7,T9}	Satisfy	Non-Functional
T9	Test Acceptance	{T7,T8}	Satisfy	Non-Functional

**Table 6:** **Requirements horizontal traceability matrix**

Requirement	MHCA-F-001	MHCA-F-002	MHCA-F-003	MHCA-F-004	MHSS-F-001
**PMCS-F-001**					
**PMCS-F-002**					
**PMCS-F-003**					
**PMCS-F-004**				Y	Y
**PMAS-F-001**					
**PMAS-F-002**					
**PMAS-F-003**					
**PMAS-F-004**				Y	Y

**Table 7:** **Test cases vs. Requirements traceability matrix**

Test Case Requirement	M1	M2	M3	M4	M5	AT1	AT2	AT3	AT4	AT5
**MHSAS-F-001**	Y						Y			
**MHSAS-F-002**	Y	Y	Y	Y	Y	Y	Y	Y	Y	Y
**MHSAS-F-003**				Y	Y			Y	Y	Y
**MHSAS-F-004**				Y	Y					Y
**MHSAS-F-005**				Y	Y	Y				Y
**MHCA-F-001**	Y						Y			
**MHCA-F-002**	Y						Y			
**MHCA-F-003**	Y						Y			
**MHCA-F-004**	Y	Y	Y	Y	Y	Y	Y	Y	Y	Y
**MHSS-F-001**	Y	Y	Y	Y	Y	Y	Y	Y	Y	Y

### Assessment

We assess our work by arguing benefit in terms of project stage with different evaluation criteria: per-assessment (initial stage of input data of modeling tools) and post-assessment with measures of soundness, applicability and project management efficiency.

- Pre-assessment

Based on our verifications, there were 122 requirements, 16 classes, 12 test cases (Integration and Acceptance) and 71 relations (refine, satisfy, etc.) between them to be examined in our research project. Fortunately, there were few errors to be discovered before inputting in our tool. The reason was unfamiliar of third-party tool. Because of there are many kinds of commercial and free open source software for modeling diagrams, although the concepts are identical. Thus, the project members need additional training before using the tool. In addition, the project scope is complex. Thus, the modeling engineer must be very careful during the analysis stage of project. Otherwise, some unusual typos and mistakes could produce during the modeling process.

b) Post-assessment
1) Soundness

We claim that our purposed work is sound in this research. From the theory perspective, the three layers model was built on the foundation of standard software engineeringapproaches. Thus, the presentation of requirement, design class, testing case in diagrams and relevantguidance of project management were shown in a standard manner with ease of understanding. Through the demonstratedcase study, the conceptual model was tested and verified to effectively represent the modeling results.

2) Applicability

The integration capabilities of our conceptual system can be utilized for working with current UML and SysML tools. We can see the successful evidences of implementation of proposed works with tools of Microsoft Visio and Excel. As shown in Tables 5, 6 and 7, the qualified results were producedand verified by the checking rules. In addition, our work can be extended to incorporate with any current UML/SysML tools as long as their products providing the data output option for diagram modeling results. In the other words, it does not depend on any specified modeling software and can be operated flexibly.

3) Project management efficiency

The (semi-) automatic approach of our work provided improvements of the efforts for managing medical software project life cycle. Based on our observation, the efforts for developing traceability between different products in different project phases can be saved. However, some efforts still require for project members in phases of requirement gathering, system analysis and design, testing and their diagram modeling processes. These works could not be archived easily because of the concerns of patient safety. In our belief, the medical staffs can use the varied tools for saving their time on these routine jobs and will result in putting more focus on the patient. Any project management tools are encouraged in our medical environment if they are safe and useful on improvements of job efficiency.

## Benefits, limits and future research

Compared with (Gurupur & Tanik 2012), our research was not only on technical perspectives, but also with different perspectives for long term project management. For example, we used general software engineering modeling standards. Thus, the modeling engineer can easily understand the project concepts of stages of analysis, development and testing. In addition, it provides insights for integration of software development theories in early stage of project implementation. More specifically, people can also integrate with varied theories to fulfill management ingredients of health IT project. In addition, project owners can use our supported tool to do change management in the project (e.g. traceability management). Thus, our research approach can be deemed as a combination of key successfully influencing factors of health IT project that we had been discussed in the literature review section.

On the other hand, (Maojo et al. 2002) considered three perspectives (Theory, Abstraction and Design) for Medical Informatics. As shown the results in our research, we demonstrated our research approach to meet components in the field. We studied and used current software engineering methodologies to create a new DCI model and experiment on Nuclear medicine project to make sure project success. In addition, the supported tool should have capabilities to resolve boundary issues of global development environments. In addition, the functions were designed and verified in our research to demonstrate a reference model on public platform to avoid integration and learning efforts on various third-party tools. It also has advantages of providing the guidance and insights to the relevant stakeholders which means they can tailor project scope by their organization needs and integrate their original management, development directions or even current popular software engineering approaches. More specifically, the practitioner and developer just follow the concepts in the reference model and most of theoriesand practices can be easily integrated into our approach without the long learning curve and additional cost. The flexibility and scalability for project long term management were considered in this approach.

However, the research has its own limits and constrains. Firstly, the CMMI-based software development standard may not familiar with software team in the project though it has been discussed since many years ago. And, the training efforts for its basic concepts are necessary. As we know, in pervious discussion, there exists management, medical and general software engineering theories. And, there should have more and more relevant development standards to be discussed or developed in the future. Thus, our approach was designed for adjusting and fitting them easily. More specifically, it provided insights for future medical software project management. Secondly, we assumed the original diagrams from third-party tools were correct. But, if the original data has some errors and are inputted in our tool, it would have unexpected results and may induce project fail. Thus, we designed the concept rules for checking such kind of mistakes. However, the traceability in the complex software project may not be understood easily. Thus, the complete and clear identification rules should be clarified and listed for automatic checking for robust modeling. In addition, the semantic interoperability of health information has been an important topic in recent years. Thus, clinical interoperability functions will be integrated in the future development version.

In sum, our research deems systematic and flexible approach is necessary. In contrast to most of the presented HIS approaches focus on their needs to present the model. In our model, we were likely to show all ingredients for successful project management. Thus, the academic researcher and industry practitioner can base on their needs to do implementation with their own software engineering methodologies and/or management technologies. In the same time, they were also given the broaden perspectives on different project phases to make sure their future project success by our research.

## Authors’ original submitted files for images

Below are the links to the authors’ original submitted files for images. Authors’ original file for figure 1 Authors’ original file for figure 2 Authors’ original file for figure 3 Authors’ original file for figure 4 Authors’ original file for figure 5 Authors’ original file for figure 6 Authors’ original file for figure 7 Authors’ original file for figure 8 Authors’ original file for figure 9

## References

[CR1] Chang Gung Medical Foundation 2012.http://www.cgmh.org.tw/eng2002/about01.htm . Accessed June 22 2012

[CR2] Chen J-J, Wang P-W, Huang Y-C, Yen H-C (2012). Applying ontology techniques to develop a medication history search and alert system in department of nuclear medicine. J Med Syst.

[CR3] Concurrent Versions System 2012.http://www.nongnu.org/cvs/ . Accessed June 11 2012

[CR4] CMMI Product Team (2010). CMMI for Development, Version 1.3 (CMU/SEI-2010-TR-033).

[CR5] Fu Y, Li M, Chen F (2012). Impact propagation and risk assessment of requirement changes for software development projects based on design structure matrix. Int J Proj Manag.

[CR6] Gagnon MP, Desmartis M, Labrecque M, Car J, Pagliari C, Pluye P, Fremont P, Gagnon J, Tremblay N, Legare F (2012). Systematic review of factors influencing the adoption of information and communication technologies by healthcare professionals. J Med Syst.

[CR7] Group OM (2012). OMG SysML.

[CR8] Gurupur V, Tanik M (2012). A system for building clinical research applications using semantic Web-based approach. J Med Syst.

[CR9] Hägglund M, Scandurra I, Koch S (2010). Scenarios to capture work processes in shared homecare—from analysis to application. Int J Med Inform.

[CR10] Hasman A, Haux R (2006). Modeling in biomedical informatics–an exploratory analysis (part 1). Methods Inf Med.

[CR11] Hasvold PE, Scholl J (2011). Flexibility in interaction: sociotechnical design of an operating room scheduler. Int J Med Inform.

[CR12] Hong CK, Guo YH, Li YC (2004). Light-weight capability maturity model integration (CMMI) for national science council (NSC) open source project. Nat Sci Council Depart Eng Applied Sci.

[CR13] Ißler L, Spreckelsen C, Weßel C (2007). Implementing software development guidelines in a medical informatics research project. Methods Inf Med.

[CR14] Leonor Teixeira C, Santos BS (2012). User-centered requirements engineering in health information systems. astudy in the hemophilia field. ComputMethods ProgramsBiomed.

[CR15] Lopez DM, Blobel BGME (2009). A development framework for semantically interoperable health information systems. IntJMed Inform.

[CR16] Mäder P, Gotel O (2012). Towards automated traceability maintenance. J Syst Softw.

[CR17] Magdaleno AM, Werner CML, Araujo RM (2012). Reconciling software development models: a quasi-systematic review. J Syst Softw.

[CR18] Maojo V, Martin F, Crespo J, Billhardt H (2002). Theory, abstraction and design in medical informatics. Methods Inf Med.

[CR19] Marcus A, Xie X, Poshyvanyk D, Marcus A, Xie X, Poshyvanyk D (2005). Proceedings of the 3rd international workshop on traceability in emerging forms of software engineering, long beach. When and how to visualize traceability links?.

[CR20] Meidani Z, Sadoughi F, Maleki M, Tofighi S, Marani A (2012). Organization’s quality maturity as a vehicle for EHR success. J Med Syst.

[CR21] Soares MS, Vrancken J, Verbraeck A (2011). User requirements modeling and analysis of software-intensive systems. J Syst Softw.

[CR22] Staples M, Niazi M, Jeffery R, Abrahams A, Byatt P, Murphy R (2007). An exploratory study of why organizations do not adopt CMMI. J Syst Softw.

[CR23] Ting S, Kwok S, Tsang A, Lee W (2011). Critical elements and lessons learnt from the implementation of an RFID-enabled healthcare management system in a medical organization. J Med Syst.

[CR24] Wendt T, Haber A, Brigl B, Winter A (2004). Modeling hospital information systems (part 2): using the 3LGM2 tool for modeling patient record management. Methods Inf Med.

