# Supplementary material for: A CMMI-based approach for medical software project life cycle study
Source: Springerplus. 2013 Jun 17;2(1):266. doi: 10.1186/2193-1801-2-266 (PMC3699709; doi:10.1186/2193-1801-2-266)
Supplement: Supplementary file 1 — Authors’ original file for figure 1 [file 40064_2013_351_MOESM1_ESM.pdf]

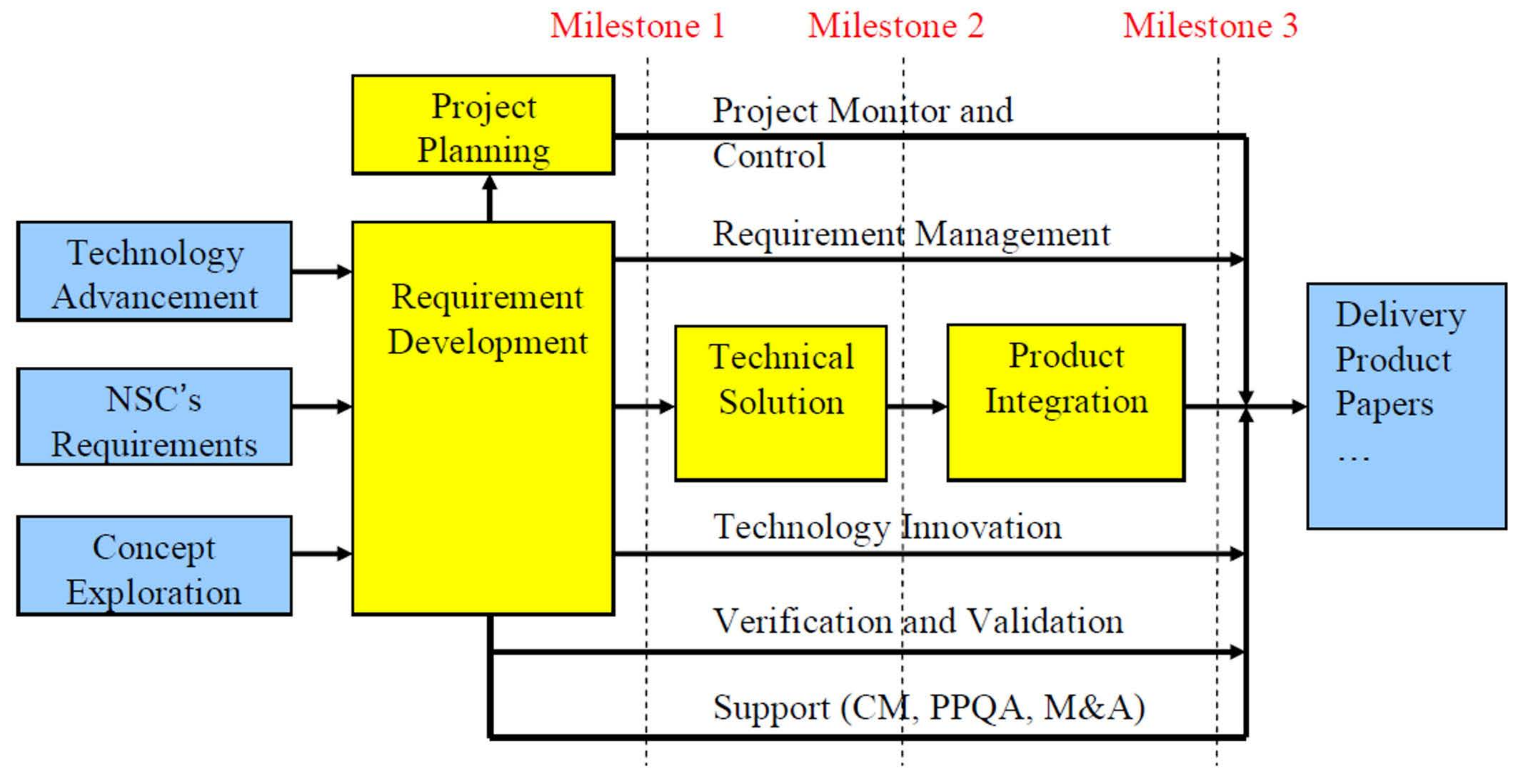
Milestone 1
Milestone 2
Milestone 3
Project Planning
Project Monitor and Control
Technology Advancement
Requirement Development
Requirement Management
NSC's Requirements
Technical Solution
Product Integration
Delivery Product Papers …
Concept Exploration
Technology Innovation
Verification and Validation
Support (CM, PPQA, M&A)
