# Supplementary material for: A CMMI-based approach for medical software project life cycle study
Source: Springerplus. 2013 Jun 17;2(1):266. doi: 10.1186/2193-1801-2-266 (PMC3699709; doi:10.1186/2193-1801-2-266)
Supplement: Supplementary file 2 — Authors’ original file for figure 2 [file 40064_2013_351_MOESM2_ESM.pdf]

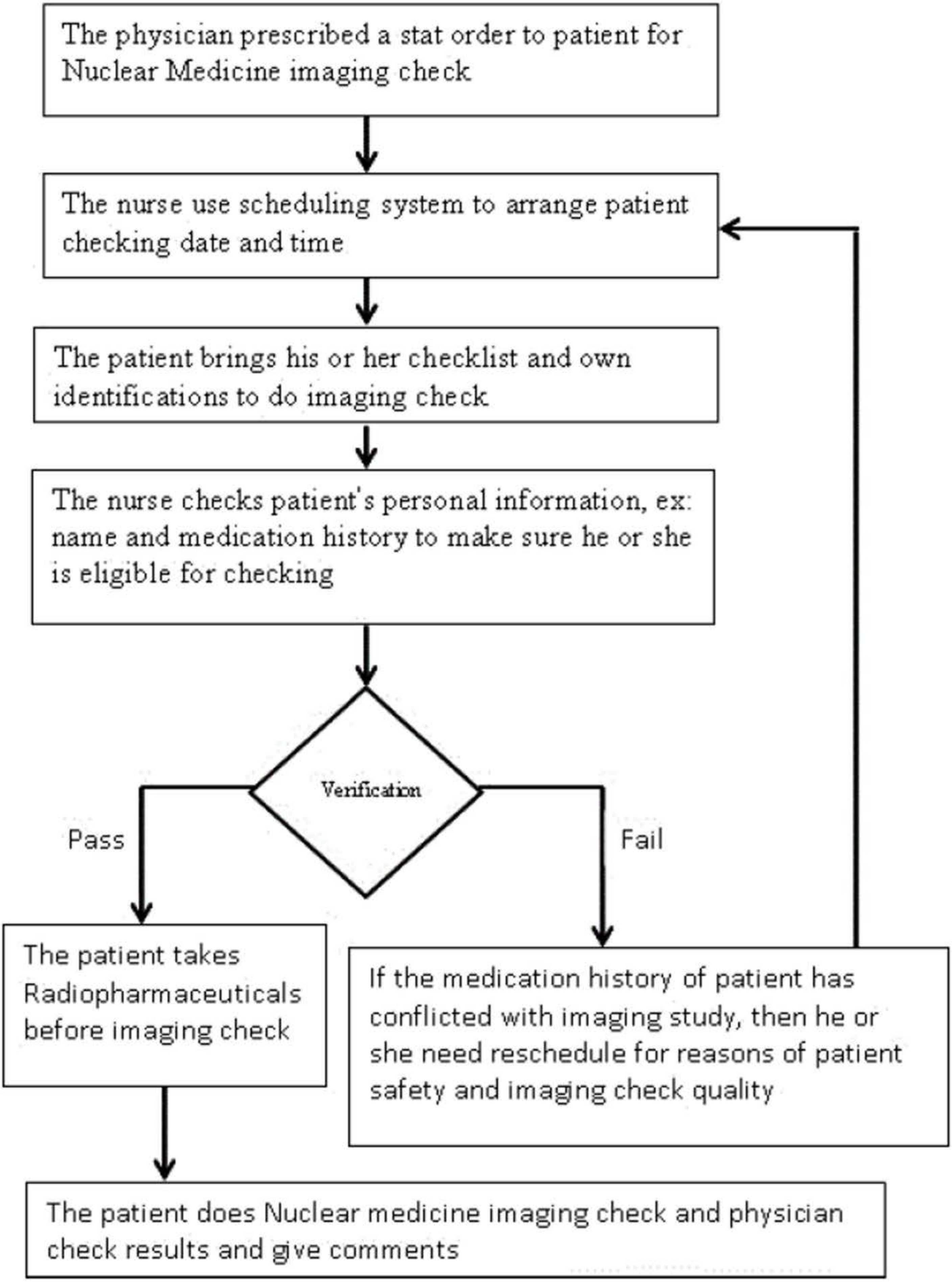
The physician prescribed a stat order to patient for Nuclear Medicine imaging check
The nurse use scheduling system to arrange patient checking date and time
The patient brings his or her checklist and own identifications to do imaging check
The nurse checks patient's personal information, ex: name and medication history to make sure he or she is eligible for checking
Verification
Pass
Fail
The patient takes Radiopharmaceuticals before imaging check
If the medication history of patient has conflicted with imaging study, then he or she need reschedule for reasons of patient safety and imaging check quality
The patient does Nuclear medicine imaging check and physician check results and give comments
