# Supplementary material for: A CMMI-based approach for medical software project life cycle study
Source: Springerplus. 2013 Jun 17;2(1):266. doi: 10.1186/2193-1801-2-266 (PMC3699709; doi:10.1186/2193-1801-2-266)
Supplement: Supplementary file 3 — Authors’ original file for figure 3 [file 40064_2013_351_MOESM3_ESM.pdf]

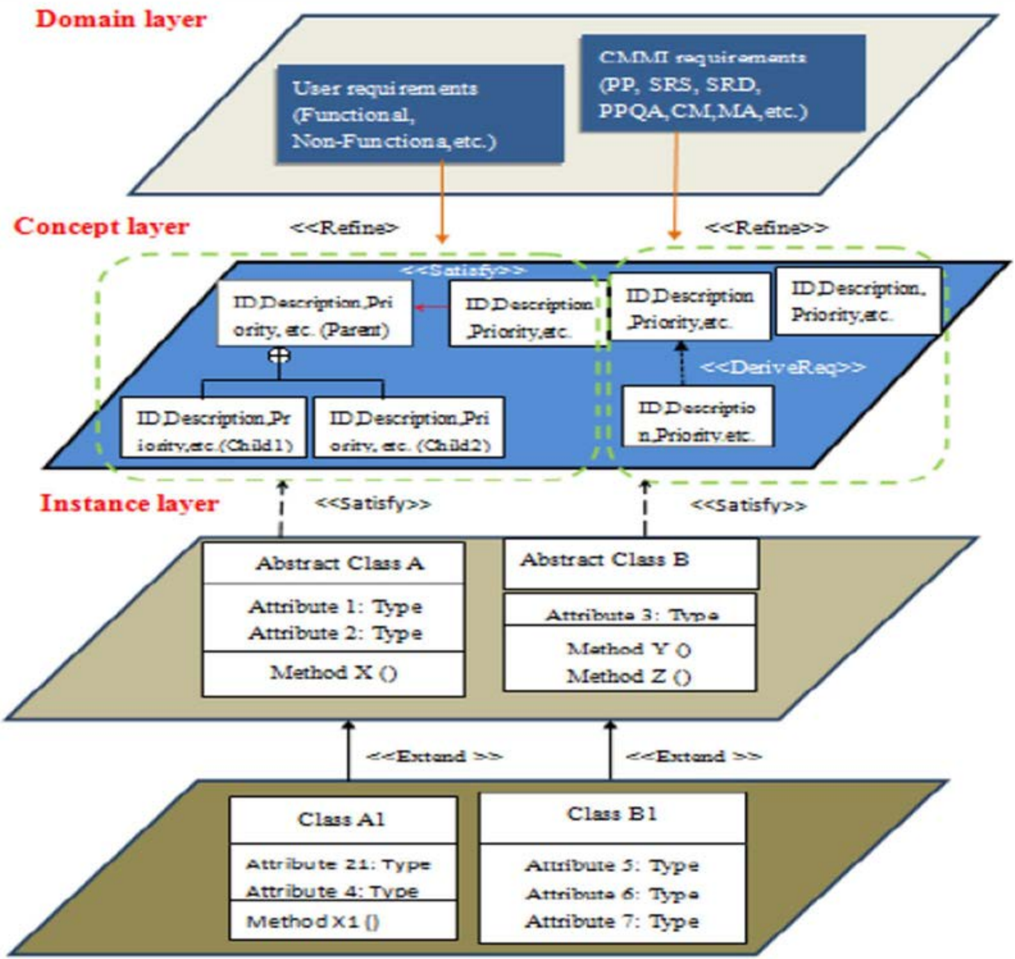

Domain layer
User requirements (Functional, Non-Functiona,etc.)
CMMI requirements (PP, SRS, SRD, PPQA,CM,MA,etc.)
Concept layer
<<Refine>
<<Refine>>
<<Satisfy>>
ID,Description,Priority, etc. (Parent)
ID,Description,Priority,etc.
ID,Description,Priority,etc.
ID,Description, Priority,etc.
<<DeriveReq>>
ID,Description,Priority,etc.
ID,Description,Priosity,etc.(Child1)
ID,Description,Priority, etc. (Child2)
Instance layer
<<Satisfy>>
<<Satisfy>>
Abstract Class A
Attribute 1: Type
Attribute 2: Type
Method X ()
Abstract Class B
Attribute 3: Type
Method Y ()
Method Z ()
<<Extend >>
<<Extend >>
Class A1
Attribute 21: Type
Attribute 4: Type
Method X1 ()
Class B1
Attribute 5: Type
Attribute 6: Type
Attribute 7: Type
