# Supplementary figures and images for: A CMMI-based approach for medical software project life cycle study
Source: Springerplus. 2013 Jun 17;2(1):266. doi: 10.1186/2193-1801-2-266 (PMC3699709; doi:10.1186/2193-1801-2-266)

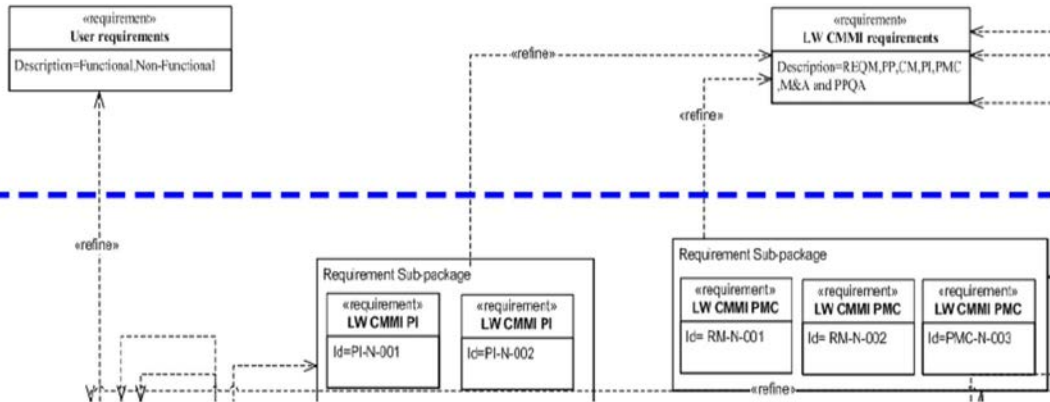

Supplement: Supplementary file 4 — Authors’ original file for figure 4 [file 40064_2013_351_MOESM4_ESM.pdf]

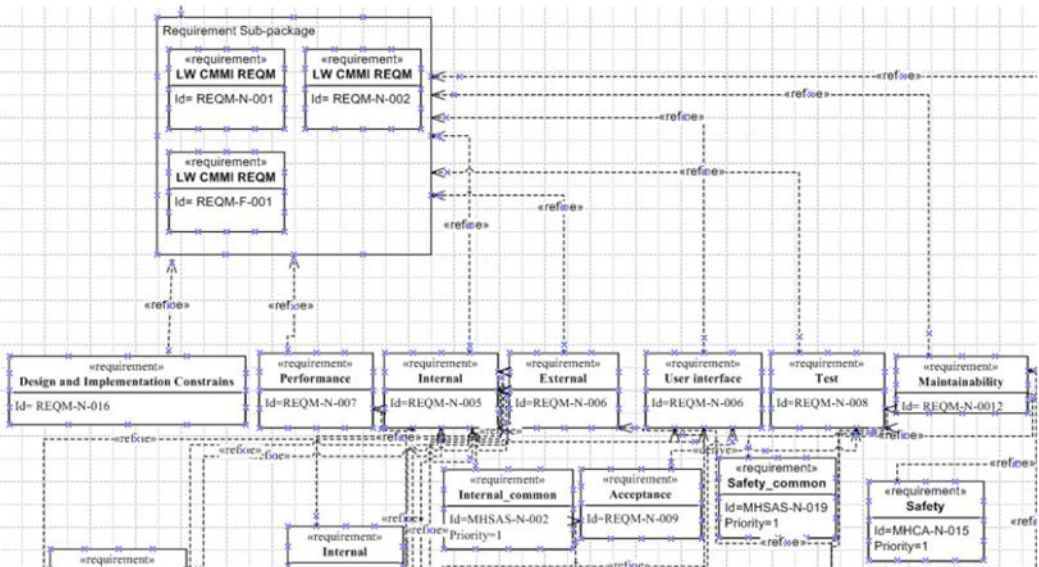

Supplement: Supplementary file 5 — Authors’ original file for figure 5 [file 40064_2013_351_MOESM5_ESM.pdf]

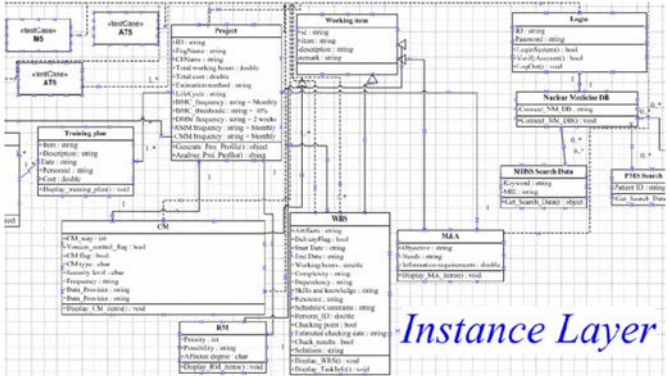

Supplement: Supplementary file 6 — Authors’ original file for figure 6 [file 40064_2013_351_MOESM6_ESM.pdf]
