# Supplementary material for: A CMMI-based approach for medical software project life cycle study
Source: Springerplus. 2013 Jun 17;2(1):266. doi: 10.1186/2193-1801-2-266 (PMC3699709; doi:10.1186/2193-1801-2-266)
Supplement: Supplementary file 7 — Authors’ original file for figure 7 [file 40064_2013_351_MOESM7_ESM.pdf]

| | A | B | C | D | E |
|---|---|---|---|---|---|
| 1 | **Id** | **Name** | **RelatesTo** | **RelatesHow** | **Type** |
| 2 | T1 | External common | {T3,T5} | DeriveReqt | Functiaonl |
| 3 | T2 | Acceptance | T4 | Satisfy | Non-Functioanl |
| 4 | T3 | Safety | T1 | DeriveReqt | Non-Functioanl |
| 5 | T4 | Internal common | T2 | DeriveReqt | Functiaonl |
| 6 | T5 | Internal common | T7 | DeriveReqt | Functiaonl |
| 7 | T6 | External common | T7 | DeriveReqt | Functiaonl |
| 8 | T7 | User-Interface | {T8,T9} | DeriveReqt | Non-Functioanl |
| 9 | T8 | Test | {T7,T9} | Satisfy | Non-Functioanl |
| 10 | T9 | Test Acceptance | {T7,T8} | Satisfy | Non-Functioanl |

**Fig 7.** The exported information by SysML tools
