# Supplementary material for: A CMMI-based approach for medical software project life cycle study
Source: Springerplus. 2013 Jun 17;2(1):266. doi: 10.1186/2193-1801-2-266 (PMC3699709; doi:10.1186/2193-1801-2-266)
Supplement: Supplementary file 8 — Authors’ original file for figure 8 [file 40064_2013_351_MOESM8_ESM.pdf]

	A	B	C	D	E	F
1	Name \ Name	MHCA-F-001	MHCA-F-002	MHCA-F-003	MHCA-F-004	MHSS-F-001
2	PMCS-F-001					
3	PMCS-F-002					
4	PMCS-F-003					
5	PMCS-F-004				Y	Y
6	PMAS-F-001					
7	PMAS-F-002					
8	PMAS-F-003					
9	PMAS-F-004				Y	Y

Fig 8. Requirements horizontal traceability matrix Table
