# Supplementary material for: A CMMI-based approach for medical software project life cycle study
Source: Springerplus. 2013 Jun 17;2(1):266. doi: 10.1186/2193-1801-2-266 (PMC3699709; doi:10.1186/2193-1801-2-266)
Supplement: Supplementary file 9 — Authors’ original file for figure 9 [file 40064_2013_351_MOESM9_ESM.pdf]

	A	B	C	D	E	F	G	H	I	J	K
1	Test Case / Requirement	**M1**	**M2**	**M3**	**M4**	**M5**	**AT1**	**AT2**	**AT3**	**AT4**	**AT5**
2	**MHSAS-F-001**	Y						Y			
3	**MHSAS-F-002**	Y	Y	Y	Y	Y	Y	Y	Y	Y	Y
4	**MHSAS-F-003**				Y	Y			Y	Y	Y
5	**MHSAS-F-004**				Y	Y					Y
6	**MHSAS-F-005**				Y	Y	Y				Y
7	**MHCA-F-001**	Y						Y			
8	**MHCA-F-002**	Y						Y			
9	**MHCA-F-003**	Y						Y			
10	**MHCA-F-004**	Y	Y	Y	Y	Y	Y	Y	Y	Y	Y
11	**MHSS-F-001**	Y	Y	Y	Y	Y	Y	Y	Y	Y	Y

Fig 9. Test cases vs. Requirements traceability matrix Table
